# Women’s perception, attitudes, and intended behavior towards predictive epigenetic risk testing for female cancers in 5 European countries: a cross-sectional online survey

**DOI:** 10.1186/s12889-019-6994-8

**Published:** 2019-05-30

**Authors:** Odette Wegwarth, Nora Pashayan, Martin Widschwendter, Felix G. Rebitschek

**Affiliations:** 1 Center for Adaptive Rationality, Max Planck Institute for Human Development, Berlin, Germany; 2 Harding Center for Risk Literacy, Max Planck Institute for Human Development, Berlin, Germany; 3 Department of Applied Health Research, Institute of Epidemiology and Healthcare, University College London, London, UK; 4 Department of Women’s Cancer, Institute for Women’s Health, University College London, London, UK

**Keywords:** Predictive epigenetic testing, Female cancer risk, European women, Attitudes, Intentions

## Abstract

**Background:**

Epigenetic markers might be used for risk-stratifying cancer screening and prevention programs in the future. Although the clinical utility of consequent epigenetic tests for risk stratification is yet to be proven, successful adoption into clinical practice also requires the public’s acceptance of such tests. This cross-sectional online survey study sought to learn for the first time about European women’s perceptions, attitudes, and intended behavior regarding a predictive epigenetic test for female cancer (breast, ovarian, cervical, and endometrial) risks.

**Methods:**

1675 women (40–75 years) from five European countries (Czech Republic, Germany, United Kingdom, Italy, Sweden), drawn from online panels by the survey sampling company Harris Interactive (Germany), participated in an online survey where they first received online leaflet information on a predictive epigenetic test for female cancer risks and were subsequently queried by an online questionnaire on their desire to know their female cancer risks, their perception of the benefit-to-harm ratio of an epigenetic test predicting female cancer risks, reasons in favor and disfavor of taking such a test, and their intention to take a predictive epigenetic test for female cancer risks.

**Results:**

Most women desired information on each of their female cancer risks, 56.6% (95% CI: 54.2–59.0) thought the potential benefits outweighed potential harms, and 75% (72.0–77.8) intended to take a predictive epigenetic test for female cancer risks if freely available. Results varied considerably by country with women from Germany and the Czech Republic being more reserved about this new form of testing than women from the other three European countries. The main reason cited in favor of a predictive epigenetic test for female cancer risks was its potential to guide healthcare strategies and lifestyle changes in the future, and in its disfavor was that it may increase cancer worry and coerce unintended lifestyle changes and healthcare interventions.

**Conclusions:**

A successful introduction of predictive epigenetic tests for cancer risks will require a balanced and transparent communication of the benefit-to-harm ratio of healthcare pathways resulting from such tests in order to curb unjustified expectations and at the same time to prevent unjustified concerns.

**Electronic supplementary material:**

The online version of this article (10.1186/s12889-019-6994-8) contains supplementary material, which is available to authorized users.

## Background

Cancer is a leading cause of mortality worldwide, accounting for 14.1 million new cases and 8.2 million deaths in 2012. [1] Prevention and early detection remain the key interventions to reduce the global cancer burden, although with modest efficacy. [2–5] Almost all cancers occur against a background of individual risk factors including genetic and nongenetic factors. In the last decade, it has been recognized that expressions of cancer-associated genes in the majority of sporadic cancers is actually controlled by deoxyribonucleic acid (DNA) methylation processes—defined as epigenomics—that can influence gene expression, without causing a permanent alteration in a gene (or DNA). The epigenome is highly dynamic. Epigenomic regulators are at work nonstop, removing or adding chemical marks that allow for transient gene readouts while blocking them in the next minute. Risk factors such as age, reproductive and lifestyle factors, and environmental exposures can trigger alterations in the epigenome, which have been implicated in the development and progression of cancer. [6] As a consequence, alterations of the epigenome have become the promising target in recent research endeavors for predicting an individual’s cancer risk; it is hoped that the information on individual’s cancer risk may help clinicians offer a more risk-tailored cancer screening and prevention management that will reduce cancer burden more effectively. In the setting of prostate cancer, for example, targeting screening to men at higher than population average risk could reduce the proportion of men likely to be overdiagnosed and, consequently, overtreated. [7, 8] Risk prediction models that incorporate epigenetic markers could thus provide new opportunities for risk stratification in risk-stratified cancer screening and prevention programs. [6]

The ongoing FORECEE (female cancer prediction using cervical omics to individualize screening and prevention) project is developing an epigenetic test to predict the risk for breast, ovarian, cervical, and endometrial cancers in women using cervical cells (https://forecee.eu). The clinical utility of this predictive epigenetic test for risk-stratification in risk-tailored cancer screening and prevention programs is yet to be assessed. However, even if in the future studies prove the test’s utility, it needs to be accepted by the public to be eventually adopted into clinical practice.

A plethora of studies exists that shed light on the public’s knowledge, attitudes, and intended behavior regarding predictive genetic testing, [9, 10] however, to date nothing is known about these matters regarding predictive epigenetic testing on cancer risk. Because, in contrast to genetic markers, epigenetic markers act as surrogate readouts for heritable and lifestyle risk factors, predictive epigenetic tests may raise questions and concerns among the public that are different from those raised by genetic testing (e.g., the “testified” individual’s responsibility for an increased risk due to a health-impairing lifestyle). A timely understanding of how the public views predictive epigenetic testing for cancer risk will therefore help to carefully guide the development of appropriate communication tools for the future.

In this study, we investigated whether European women (i) perceive the potential benefits of predictive epigenetic testing for female cancer risks to outweigh the potential harms or vice versa, (ii) want to know their risk for female cancers, (iii) have personal reasons in favor and in disfavor of such a test, (iv) intend to use such a test if available, and (v) whether these perceptions, attitudes, and intended behaviors vary by country.

## Methods

### Study oversight

The study was set up as a cross-sectional population-based online survey with women from five European countries that represent Northern, Eastern, Southern, Western, and Central Europe and the nationalities of members of the FORECEE consortium. Based on these criteria the countries chosen were the Czech Republic, Germany, United Kingdom (UK), Italy, and Sweden. The design and content of the study was developed by the authors, discussed in detail with country-specific clinical partners of the FORECEE consortium (https://forecee.eu), and revised after feedback. To execute the study online, the survey sampling company Harris Interactive (Hamburg, Germany) was contracted in order to conducted the online study—including an online version of a leaflet and an online questionnaire—by using their online panels and the online panels of Toluna, respectively. Both online panels—comprising about 78,000 and 275,000 online panelist, respectively—are representative of the general population in these countries.

The study was performed in accordance with relevant guidelines and regulations, and informed consent was obtained from all participants prior to the study. The study was approved by the independent Institutional Ethics Review Board of the Max Planck Institute for Human Development (Germany).

### Sampling procedure and study sample

The goal of the online study was to survey national samples of women who will likely be the target group for future predictive epigenetic tests for female cancer risks (40 to 75 years) in the Czech Republic, Germany, UK, Italy, and Sweden. To reduce nonrespondent bias and to better reflect the general population of women of the target group in each country, the samples were stratified based on the official combined distribution of age and education per country at the point of survey completion. Quotas per country were drawn from EUROSTAT (European Statistical System) [11] and calculated on the grounds of three levels of education (low, medium, high) as categorized by the International Standard Classification of Education (ISCED) and for four age groups (40–49 years, 50–59 years, 60–69 years, 70–75 years). We calculated that a sample size of about 300 participants per country was required to detect differences in perceptions and intended behavior (2-sided alpha of .05) of 10% or higher with a 90% power between the national samples. To allow for nonresponse and ineligibility, in January 2017, Harris Interactive sent invitation links by email to those of their female online panelists who matched the selection criteria in age and nationality. In case, the invitation caught the interest of an online panelist, she clicked on a link provided in the invitation email that directed her to the online portal of Harris Interactive. After providing informed consent, women entered the online study—entirely hosted by Harris Interactive—which included both the online survey questionnaire and the online leaflet. After study completion, the authors of this study received a completely anonymized data sheet from Harris Interactive. Thus, all respondents of this study are fully anonymous to the authors.

Of 3629 women contacted by Harris Interactive, 848 did not respond, 197 were not eligible (duplicate listing, other strata than originally coded), and 492 entered the online study after respective quotas were filled. Of the 2092 who responded, 417 did not finish the online study, resulting in 1675 completed online surveys. Using the AAPOR (American Association for Public Opinion Research) response rate calculator, [12] which incorporates a default method for estimating *e* (estimated proportion of cases of unknown eligibility that is eligible), the study yielded a response rate of 61.4% (1675/[1675 + 417 + *e* (848)]) and a cooperation rate of 80.1% (1675/[1675 + 417]).

Of the 1675 online surveys collected in total, 356 were completed in the Czech Republic, 335 in Germany, 323 in the UK, 338 in Italy, and 323 in Sweden. Across countries, the distribution of age groups was 40–49 years (29.6%), 50–59 years (29.3%), 60–69 years (29.5%), and 70–75 years (11.7%) and the distribution of educational levels was low (27.8%), medium (48.8%), and high (23.5%). 157 women reported a personal history of any cancer in the past and 104 specifically of female cancers. Table 1 provides country-specific details on these characteristics.

**Table 1:** Characteristics of the study sample

Number of participants (%)													
	All countries (*N* = 1675)		Czech Republic (*n* = 356)		Germany (*n* = 335)		United Kingdom (*n* = 323)		Italy (*n* = 338)		Sweden (*n* = 323)		*p*-value*
Age group (years)													.090
40–49	495	(29.6%)	100	(28.1%)	96	(28.7%)	101	(31.3%)	102	(30.2%)	96	(29.7%)	
50–59	490	(29.3%)	90	(25.3%)	109	(32.5%)	101	(31.3%)	96	(28.4%)	94	(29.1%)	
60–69	494	(29.5%)	131	(36.8%)	82	(24.5%)	82	(25.4%)	101	(29.9%)	98	(30.3%)	
70–75	196	(11.7%)	35	(9.8%)	48	(14.3%)	39	(12.1%)	39	(11.5%)	35	(10.8%)	
Education (ISCED)													<.001
High	393	(23.5%)	52	(14.6%)	65	(19.4%)	116	(35.9%)	43	(12.7%)	117	(36.2%)	
Medium	817	(48.8%)	256	(71.9%)	207	(61.8%)	111	(34.4%)	112	(33.1%)	131	(40.6%)	
Low	465	(27.8%)	48	(13.5%)	63	(18.8%)	96	(29.7%)	183	(54.1%)	75	(23.2%)	
Personal history of female cancer													.829
Yes	104	(6.2%)	20	(5.6%)	23	(6.9%)	19	(5.9%)	20	(5.9%)	22	(6.8%)	
Unknown	25	(1.5%)	6	(1.7%)	1	(0.3%)	4	(1.2%)	7	(2.1%)	7	(2.2%)	
No	1546	(92.3%)	330	(92.7%)	311	(92.8%)	300	(92.9%)	311	(92.0%)	294	(91.0%)	

*p-values derived from Chi-Square analysis

Respondents in each of the national online samples were similar to their respective general population in terms of the distribution of age and education, with the following exceptions: For women from the Czech Republic and Sweden presenting with low education, the age group “60 to 69 years” were overrepresented and “70 to 75 years” were underrepresented (Additional file 2: Table S1).

### Information leaflet and survey questionnaire

To ensure that all women were equipped with sufficient background information on predictive epigenetic testing for female cancer risk, the study was designed as such that the women entering the online study were first presented with an online leaflet—whose content they were advised to familiarize themselves with—before being subsequently presented with the online survey questionnaire. While then working through the online survey questionnaire, women could easily re-approach the content of the online leaflet at any time. The online leaflet (see Additional file 2) included details on the following aspects:

- Estimates of age-adjusted risks of breast, ovarian, cervical, and endometrial cancer
- Current approaches of cancer screening and prevention with examples of the benefit-to-harm ratio
- Rationale behind predictive epigenetic testing for female cancer risks and its core principles
- Potential benefit and harms of such predictive epigenetic testing
- Additional information (e.g., confidentiality of data, state of evidence)

The development of the online survey questionnaire—designed to elicit women’s perception, attitudes, and intended behavior towards predictive epigenetic testing for female cancer risk—was informed by an assessment of findings published on these aspects in the field of genetic testing, in-depth discussions with the clinical partners and (epi-)genetic experts of the FORECEE consortium group, and by outcomes of focus group discussions with 25 women from Germany and 12 women from the UK. The survey questionnaire (for the exact wording, see Additional file 2) first asked four questions on the core principles of predictive epigenetic testing to ensure that women had a sufficient basic understanding of the cancer types targeted by the test, the potential impact of different test outcomes, and the interplay between external factors and the epigenome. For each of these four questions, women were presented with a choice of three answers of which one was correct. Women’s perception of the benefit-to-harm ratio of predictive epigenetic testing for female cancer risks was measured by a 5-point-Likert scale reaching from “harms clearly outweigh potential benefits” to “benefits clearly outweigh potential harms.” Their desire to learn about their cancer-specific risk for each of the four cancer types and their intention to take predictive epigenetic testing for female cancer risks if the test were freely available were measured by a binary choice (yes/no). To explore reasons in favor or disfavor of the test, we presented women with lists of items in favor (e.g., motivate the adoption of healthier lifestyle) and disfavor (e.g., induce unnecessary worry) of predictive epigenetic testing that were derived from focus group discussions with women in Germany and the UK. Women were asked to tick the reasons that would personally matter to them and assign ranks of importance to each (assignment of equal ranks to different reasons was possible). After women had made their choices on reasons in favor and disfavor, each woman was again presented with her chosen reasons and asked if one of these reasons was so strong that it would outweigh all the other reasons in favor or in disfavor of the test.

All study materials (online survey questionnaire, online survey leaflet) were translated into country-specific languages by a professional translation office and checked for correctness and completeness by country-specific members of the FORECEE consortium. The survey sampling company Harris Interactive (Hamburg, Germany) programmed the online version of the survey.

### Outcomes and analysis

The online version of the questionnaire did not allow for item nonresponse, thus all 1675 questionnaires were completed. Primary outcome measures were (i) women’s evaluation of the benefit-to-harm ratio of a predictive epigenetic test for female cancer risks, (ii) women’s desire to know their cancer risk per cancer site, (iii) women’s reasons in favor of having predictive epigenetic testing for female cancer risk, (iv) women’s reasons in disfavor of having predictive epigenetic testing for female cancer risk, and (v) women’s intention to use predictive epigenetic testing for female cancer risks. All results on the primary outcomes were calculated as absolute proportions with 95% confidence intervals.

Binary and multivariate logistic regression models were used to investigate whether differences between countries for each of the primary outcomes were confounded by covariates such as women’s knowledge of the core principles of predictive epigenetic testing, their age, their education, or their personal cancer history. If not reported otherwise, none of these variables significantly influenced country-specific differences. Bonferroni corrections were employed to correct for multiple testing, and *α* was set at 0.05/5 = .01. All data were stored and analyzed with IBM SPSS Statistics 24 (New York City, USA). To facilitate clear and unbiased reporting of our study results, the STROBE (Strengthening the Reporting of Observational studies in Epidemiology) reporting guideline was applied (Additonal file 1: STROBE checklist). 

## Results

### Women’s knowledge of core principles of the predictive epigenetic test for female cancer risk

Overall, between 57.4% (55.0, 59.8) and 94.6% (95% confidence interval [CI]: 93.4, 95.6) of women provided correct responses to each of the four questions on the core principles of the predictive epigenetic test for female cancer risk (Table 2). Women were least likely to correctly respond to the question on how a lower-than-average risk test result may guide their cancer screening and prevention management. Women from the Czech Republic (*p* < .001) and women with lower education (*p* < .001) were more likely to provide lower proportions of correct responses on core principles than women from the other European countries or women with higher education (Additional file 2: Table S2). After adjusting for education in multivariate analyses and multiple testing, the significant association between country and women’s knowledge of core principles (*p* = < .001) remained.

**Table 2:** Proportion of women’s correct responses on the questions of the core principles of predictive epigenetic testing for breast, ovarian, cervical, and endometrial cancer risk

	Percentage of correct responders (95%CI)						
Correct items of the surveyed core principles of predictive epigenetic testing for female cancer risks	All Countries *N* = 1675	Czech Republic *n* = 356	Germany *n* = 335	United Kingdom *n* = 323	Italy *n* = 338	Sweden *n* = 323	*p*-value*
The predictive epigenetic test for female cancer risks targets a woman’s risk for breast, ovarian, cervical, and endometrial cancer.	94.6 (93.4, 95.6)	93.5 (90.5, 95.9)	98.5 (96.6, 99.5)	96.0 (93.2, 97.8)	89.1 (85.2, 92.2)	96.0 (93.2, 97.8)	<.001
With a lower-than-average risk result, a woman could consider having less screening and consequently may reduce her likelihood of overdiagnosis and false alarms.	57.4 (55.0, 59.8)	43.0 (37.8, 48.3)	57.0 (51.5, 62.4)	81.1 (76.4, 85.2)	62.1 (56.7, 67.3)	44.9 (39.5, 50.5)	<.001
With a higher-than-average risk result, a woman could consider having more screening or preventive measure in order to reduce her likelihood of dying from cancer.	76.2 (74.1, 78.2)	47.5 (42.2, 52.8)	83.9 (79.5, 87.7)	87.6 (83.5, 91.0)	76.3 (71.4, 80.8)	88.2 (84.2, 91.5)	<.001
Our environment and lifestyle is changing the epigenome of our cells.	75.3 (73.2, 77.4)	66.3 (61.1, 71.2)	79.1 (74.4, 83.3)	87.3 (83.2, 90.7)	72.8 (67.7, 77.5)	72.1 (66.9, 77.0)	<.001

*p-values derived from Chi-Square analysis

### Women’s evaluation of the benefit-to-harm ratio of the predictive epigenetic test

**Fig. 1:**
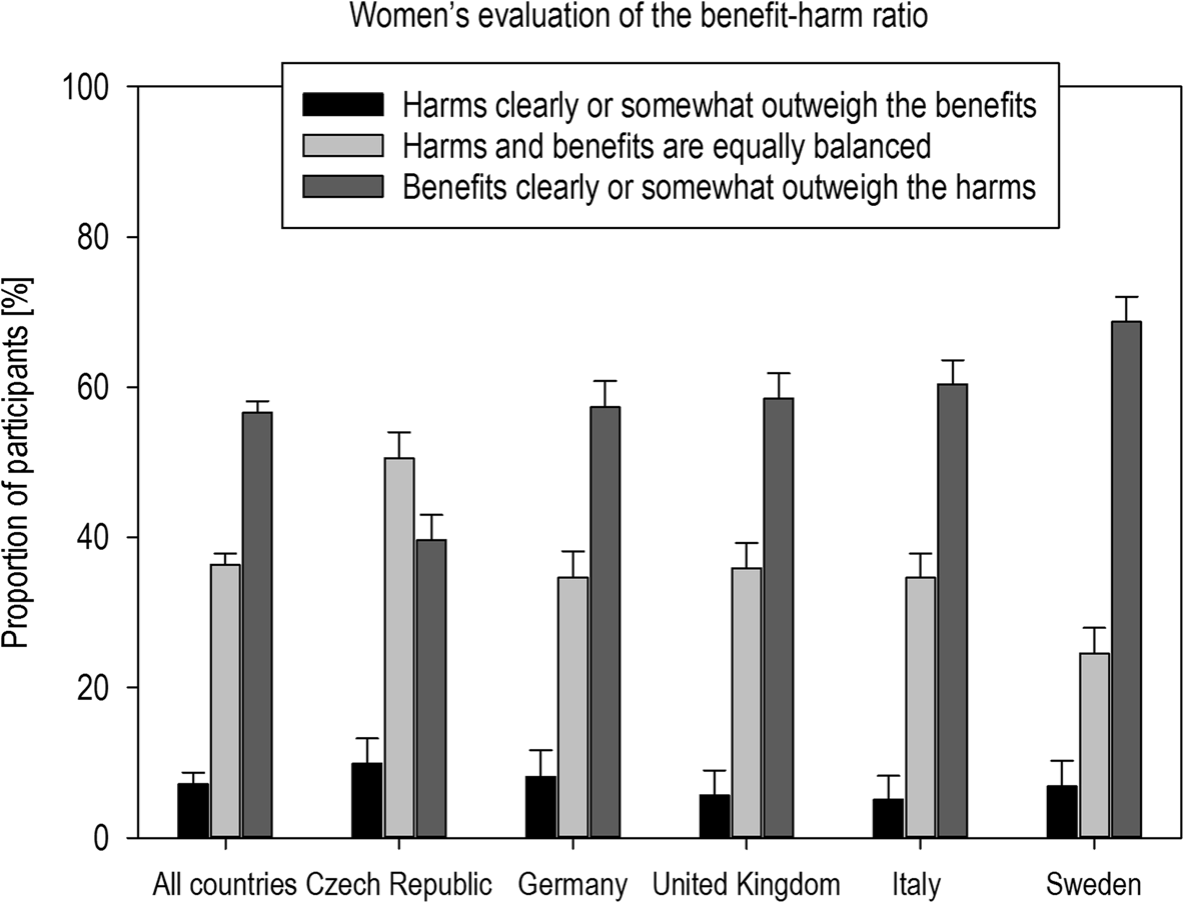
Distribution of women’s evaluation of the benefit-harm-ratio of a predictive epigenetic test for female cancer risks with standard errors

More than half (56.6, 95% CI: 54.2, 59.0) of all women felt that the potential benefits of a predictive epigenetic test for female cancer risks would somewhat or clearly outweigh its potential harms. This evaluation was shared by the majority of women within each of the studied European countries, except by women from the Czech Republic where the majority thought that the test’s harms equal its benefits (*p* < .001) (Fig. 1). After adjusting for covariates (knowledge of core principles, education, personal female cancer history, age) and multiple testing, compared to women from the Czech Republic, women from the other four countries were on average only about half as likely to consider the harms of a predictive epigenetic test for female cancer risks to equal or to somewhat/clearly outweigh the benefits (Additional file 2: Table S2).

Also, women with limited knowledge of the test’s core principles (≤50% correct) evaluated the test more negatively in univariate analyses (*p* < .001). Multivariate analysis showed, however, that the association between women’s benefit-to-harm evaluation and their knowledge of the test’s core principles was confounded by their level of education (*χ*^*2*^ (1) = 22.48; *p* < .001). After adjusting for covariates and multiple testing, women presenting with limited knowledge of the test’s core principles were seen to be nearly 2 times as likely (odds ratio [OR]: 1.79, 95% CI: 1.24–2.25, *p* < .001) to consider the harms of the predictive epigenetic test to equal or somewhat/clearly outweigh the benefits than women presenting with higher degrees of knowledge (> 50%) (Additional file 2: Table S2).

### Women’s desire to know their female cancer risks

The majority of women (63.6%) said they want to know their individual risk for each of the four female cancers (breast, ovarian, cervical, endometrial) (Table 3). Within each country, women’s desire to know the risk for one female cancer over the other cancers did not differ significantly. Women’s desire differed between countries, however: Compared to women from the Czech Republic, women from the UK, Italy, and Sweden were about 2 to 3 times more likely to desire risk information on three or all female cancers in multivariate analysis (Additional file 2: Table S2), whereas women from Germany did not differ from the Czech Republic (*p* = .122). Also, women’s age was associated with a higher desire to learn about the risk for female cancers in univariate and multivariate analysis, with women aged 40 to 59 years being significantly more likely to request such predictive information on their cancer risk than women older than that (Additional file 2: Table S2).

**Table 3:** Women’s desire to know their 10-year risk of developing breast, ovarian, cervical, and endometrial cancer

	Percentage of responders who said they want to know the cancer risk (95% CI)						
Want to know their risk for …	All Countries *N* = 1675	Czech Republic *n* = 356	Germany *n* = 335	United Kingdom *n* = 323	Italy *n* = 338	Sweden *n* = 323	*p*-value*
Breast cancer	71.7 (69.5, 73.8)	65.2 (60.0, 70.1)	58.2 (52.7, 63.5)	76.8 (71.8, 81.3)	83.4 (79.0, 87.2)	75.5 (70.5, 80.1)	<.001
Ovarian cancer	66.6 (64.3, 68.9)	57.6 (52.3, 62.8)	52.8 (47.3, 58.3)	71.5 (66.3, 76.4)	80.8 (76.2, 84.8)	71.2 (65.9, 76.1)	<.001
Cervical cancer	65.7 (63.3, 67.9)	58.1 (52.8, 63.3)	50.7 (45.3, 56.2)	70.0 (64.6, 74.9)	80.5 (75.8, 84.6)	69.7 (64.3, 74.6)	<.001
Endometrial cancer	65.0 (62.7, 67.3)	55.6 (50.3, 60.9)	51.0 (45.6, 56.5)	68.7 (63.4, 73.7)	80.2 (75.5, 84.3)	70.3 (65.0, 75.2)	<.001

*p-values derived from Chi-Square analysis

### Women’s reasons in favor of a predictive epigenetic test for female cancer risks

**Fig. 2:**
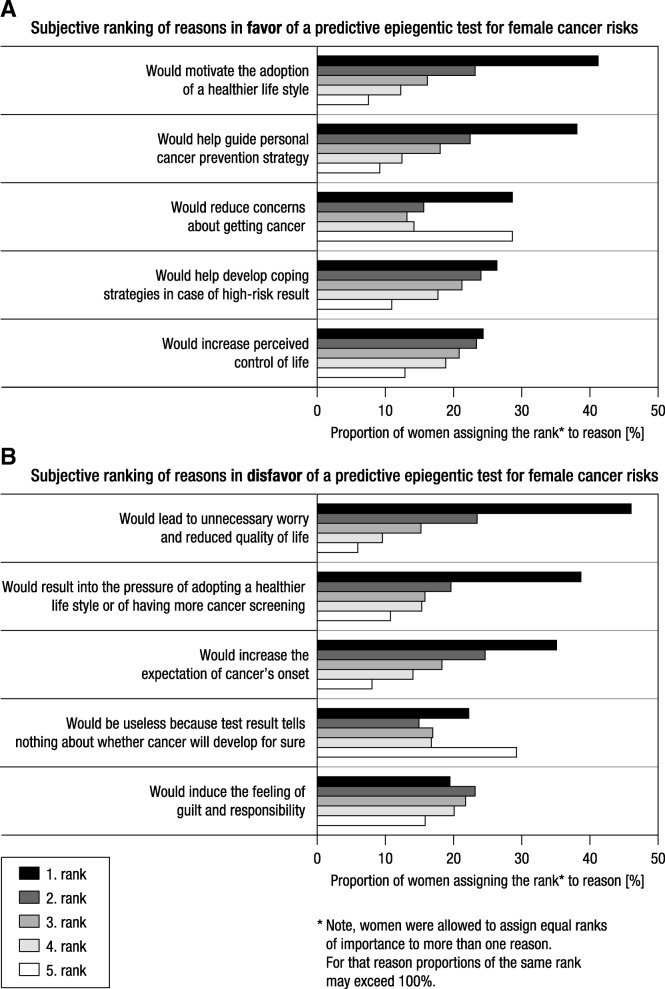
Women’s subjective ranking of the importance of reasons in (**a)** favor and (**b**) disfavor of a predictive epigenetic test for female cancer risks (data are pooled across countries)

When women were presented with the five potential reasons in favor of taking this epigenetic test, 67.2% of all women chose all five reasons, 3.9% chose four reasons, 6.4 and 7.3% chose three and two reasons, respectively, and 15.3% chose one reason. Figure 2a shows women’s ranking of their perceived importance of each of the reasons in favor of the test. When subsequently presented with all individually ticked reasons, 817 (48.8%) out of 1675 women indicated that one of these reasons was so strong that it outweighed all the others. Among these, the test’s potential to guide the personal cancer prevention strategy was the most decisive reason for women in Germany (38.8%), the Czech Republic (29.1%), and Italy (30.9%), whereas for women in the UK (37.0%) and Sweden (30.4%), it was its potential to motivate the adoption of a healthier lifestyle.

### Women’s reasons in disfavor of the predictive epigenetic test for female cancer risks

When presented with the five reasons that might speak in disfavor of taking the predictive epigenetic test, 61.1% of all women chose all five reasons, 3.5% chose four reasons, 5.6 and 8.6% chose three and two reasons, respectively, and 21.3% chose one reason. Figure 2b shows women’s ranking of their perceived importance of each of the reasons in disfavor of the test. When again subsequently presented with all individually ticked reasons in disfavor of the test, 884 (52.8%) out of 1675 women indicated that one of these reasons was so strong that it outweighed all the others. That the result of the test may induce unnecessary worry and reduce quality of life was the most decisive reason against taking the test for women in the UK (46.7%) and Sweden (33.5%), whereas for women in Germany (37.1%) and Italy (30.9%) it was the test’s potential to cause a woman to permanently expect the onset of cancer. Women in the Czech Republic (39.4%), in contrast, were most worried about the fact that the test result may pressure them into adopting a healthier lifestyle or to undergo more cancer screening.

### Women’s intention to take the test if it was already available

Overall, 75% of women responded that they would definitely or probably take this epigenetic test if it were freely available. However, women’s preparedness to take the test depended on the country they came from (*p* < .001) and their initial evaluation of the test’s benefit-to-harm ratio (*p* < .001). After adjusting for covariates and multiple testing, women from the Czech Republic, UK, Italy, and Sweden were about 2 to 3.5 times more likely to consider taking the test if it were freely available than women from Germany (Additional file 2: Table S2). Results were confounded, however, by women’s evaluation of the test’s benefit-to-harm (*χ*^*2*^ (1) = 92,67; *p* < .001), with women evaluating the test rather negatively being less prepared to take the test (*p* < .001). In accordance with this finding, we found that women who intended to take the test were also more likely to tick four or all five reasons in favor of the test than women who said they would definitely or probably not take the test: 73.7% (71.1, 76.1) versus 63.3% (58.4, 67.9). Likewise, women who indicated that they would not take the test were more likely to tick four or all reasons in disfavor of the test than women who indicated they would take the test: 71.9% (67.3, 76.2) versus 62.5% (59.8, 65.2).

## Discussion

Of the 1675 European women surveyed in our study, almost three quarters demonstrated sufficient knowledge (≥75% correct responses) of the core principles of a predictive epigenetic test for female cancer risks after reading the leaflet. Within each country, women were least likely to know, however how the prediction of a lower-than-average risk for female cancer may impact their future cancer screening and prevention management. We can only speculate about why women particularly struggled with answering this question. For one, the idea of having less screening may have conflicted with the conventional wisdom that screening always means “early caught, successfully fought.” Information that diverges from established opinion or attitudes is likely to be ignored. [13] Also, research showed that the concepts of “overdiagnosis” and “overtreatment” in cancer screening are hardly known by most people, [14, 15] which is why some women in our survey may have found it harder to memorize this unfamiliar information. [16] The knowledge of core principles also varied between countries, with women from the Czech Republic being least likely to correctly answer the questions. The observed variation was confounded by women’s education, where those with a high degree of education were almost 3 times as likely to know at least 75% of the core principles of the test than women with a low degree of education.

Women’s knowledge of the core principles had considerable consequences on how women evaluated the benefit-to-harm ratio of the predictive epigenetic test on cancer risks: While the majority of women in each country—except women from the Czech Republic— viewed the benefit-to-harm ratio positively, women with limited knowledge (≤50%) of the test’s core principles were likely to see the harms prevailing. However, this association between women’s evaluation and their knowledge of the test’s core principles was confounded by education. Given that women’s evaluations significantly influenced women’s intention to take a predictive epigenetic test, this finding matters. If women with lower levels of education decide against having a potentially effective test (once these are available and have been proven effective on the basis of solid evidence) because they do not understand the related health information, then health disparity may occur. Already now, few leaflets on cancer screening, letters of invitation to screenings, and health websites provide balanced information on screenings’ benefit and harms (e.g., as absolute risk information) that would enable women to make an informed choice. [17–21] Instead, they often refer to relative statistics, lifetime incidences, 5-year survival rates, or no numbers at all, all of which contribute to women seriously overestimating their own cancer risk and the benefit of screening and underestimating the harms. [22–26] To avoid unreasonably high expectations but also unfounded misgivings about the new generation of predictive epigenetic tests, health policy makers and health care providers need to ensure that clear principles for the reporting on future predictive epigenetic testing are in place before the tests enter the health care system. First guidance for such transparent risk communication following established reporting principles is already available. [27–31]

Our survey further sheds light, for the first time, on specific reasons that drive a positive or negative evaluation. On the positive side, women appreciate that predictive epigenetic tests may help to improve their cancer screening and prevention management and, specifically for epigenetic testing, may even motivate the adoption of a healthier lifestyle. On the negative side, they well perceived that receiving a predictive epigenetic test result could induce permanent worries about cancer and pressure them to engage in lifestyle changes that they had not intended to adopt. Considering that professionals in the health care system (e.g., gynecologists) are envisaged to provide the epigenetic tests for predicting female cancer risks, it will be particularly the responsibility of health care professionals to address any ambiguity about the test by transparently and sufficiently counsel women about the test’s accuracy, the meaning of risk-stratified test results on individual care, and their impact on the individual’s life and health-related outcomes. Given the number of studies that document health professionals’ difficulties in understanding and communicating medical risks [32–37] and test statistics [38–40], clear principles for the counselling on future predictive epigenetic testing need to be also established in training programs tailored to the healthcare professionals involved in predictive epigenetic testing. The apparent desire of many women to learn more about their cancer risk by epigenetic risk prediction [41–43] may offer new and more efficient pathways in the fight against cancer but also means that the existing communicative and educative problems documented in cancer screening and prevention need first to be resolved.

Finally, our survey revealed some country-specific differences that are hard to explain and about which we can only speculate. For instance, women from Germany were least likely to express their intention to have the predictive epigenetic test on cancer risks, while at the same time expressing a rather positive evaluation of the benefit-to-harm-ratio and presenting with sufficient knowledge of the core principals. One reason for this observation might be rooted in how German media cover personalized medicine. Whereas German media provide a relatively conservative view on such new technology and also point to its potential shortcomings, media in the UK, for instance, highlight its potential merits. [44] We also found country-specific variations in Europeans women’s knowledge of the core principles, particularly for the questions that interrogated how lower−/higher-than-average risk results may affect cancer screening and prevention uptake. Part of this variation might be influenced by women’s current knowledge and adherence to individual country policies on screening. All of the five European countries have implemented the same population-based screening programs for both breast and cervical cancers but differ in terms of organizational characteristics (e.g., standardized informational material) and implementation stage. For instance, women in Germany and the UK receive a standardized leaflet on benefit and harms of mammography when being invited to screening, but women in the Czech Republic and Italy do not. Also, the intervals of screening differ across countries, such as a 2-year interval in Germany and a 3-year interval in the UK. However, it goes beyond the scope of this study to examine to what extent these variations in information politics and implementation stages influence the reception of information on new test opportunities and which cognitive, emotional or structural mechanisms are in place; this question needs to be clarified in future studies.

Our findings need to be viewed in the light of some limitations. First, we measured women’s intention to take a predictive epigenetic test and not their actual behavior. Previous research demonstrated modest correspondence between people’s reported intention and their actual behavior—known as intention-behavior-gap. [45] It is thus likely that the observed intention to take a predictive epigenetic test for female cancer and the potential actual uptake of such tests in the future will differ from one another. Second, we used an epigenetic risk prediction test that is currently under development (Women’s identification test/WID test) for four female cancers as an example for introducing women to such future test opportunities in the field. Due to its developmental state, we were not able to provide women with any numerical estimates on the clinical utility of the test and the likely benefit-to-harm ratios of epigenetic risk-tailored cancer screening and prevention programs. Such numbers may have had a significant impact on women’s evaluation of the benefit-to-harm ratio and their reported intention to take such a test. Third, because women in our survey were not offered the option to include any further reasons in favor and disfavor for predictive epigenetic testing than the five reasons offered, we cannot exclude the possibility that reasons other than displayed in our survey have played a role in women’s evaluation of such a test. Fourth, we cannot rule out the existence of a nonrespondent bias. Although we achieved a reasonable response rate and were able to roughly match women’s characteristics for age and education in the national samples to the general population at survey completion, we cannot exclude the likelihood that women with a higher-than-average cancer risks due to close family history or those with a greater interest in the topic of cancer and cancer screening were more likely to respond to our survey, which might have influenced and limited the generalizability of our results.

## Conclusion

Despite these limitations, our survey is the first to show how European women evaluate the opportunity to have their female cancer risks predicted by an epigenetic test, what this evaluation depends on, and how this evaluation may influence the acceptance of these tests in the future. Women who are not in a position to sufficiently understand the actual impact of future predictive epigenetic tests on their healthcare might be likely to discard a potential viable test opportunity. Their understanding of and trust in this new technology will largely depend on how healthcare professionals and healthcare providers inform and counsel women in the future. [46] A balanced and transparent communication of the chances and risks of predictive epigenetic tests will be required in order to curb undue hopes but also prevent unjustified concerns about these forthcoming testing opportunities.

## Additional files

Additional file 1: STROBE checklist. (DOC 98 kb)

Additional file 2: Survey questionnaire. **Table S1.** Joint official distribution for age and education (ISCED levels) in base population per country and joint distribution within each of the national samples of the study. **Table S2.** Multivariate logistic regression for predictors of European women’s knowledge of core principals of epigenetic predictive testing for female cancer risk, their evaluation of the benefit-to-harm ratio of such tests, their desire to know their female cancer risks, and their intention to take epigenetic predictive testing for female cancer risks. Survey leaflet. (PDF 557 kb)

## Abbreviations

CI: Confidence interval
DNA: Deoxyribonucleic acid
FORECEE: Female cancer prediction using cervical omics to individualise screening and prevention
OR: Odds ratio
UK: United Kingdom of Great Britain and Northern Ireland
